# Experience and social factors influence movement and habitat selection in scimitar-horned oryx (*Oryx dammah*) reintroduced into Chad

**DOI:** 10.1186/s40462-022-00348-z

**Published:** 2022-11-10

**Authors:** M. M. Majaliwa, L. F. Hughey, J. A. Stabach, M. Songer, K. Whyle, A. E. A. Alhashmi, M. Al Remeithi, R. Pusey, H. A. Chaibo, A. Ngari Walsoumon, M. Hassan Hatcha, T. Wacher, C. Ngaba, J. Newby, P. Leimgruber, K. Mertes

**Affiliations:** 1 Conservation Ecology Center, Smithsonian’s National Zoo and Conservation Biology Institute, Front Royal, VA USA; 2 School of Mathematics and Statistics, University of Glasgow, Glasgow, G12 8QQ UK; 3 Terrestrial and Marine Biodiversity, Environment Agency, Abu Dhabi, United Arab Emirates; 4 Direction de la Conservation de la Faune et des Aires Protégées, N’Djamena, Chad; 5 SaharaConservation, Saint-Maur-des-Fossés, France; 6 Conservation Programmes, Zoological Society of London, London, UK

**Keywords:** Reintroduction, Integrated step selection functions, Social learning, Post-release experience, Seasonality, Monitoring

## Abstract

Reintroduced animals—especially those raised in captivity—are faced with the unique challenge of navigating a wholly unfamiliar environment, and often make erratic or extensive movements after release. Naïveté to the reintroduction landscape can be costly, e.g., through increased energy expenditure, greater exposure to predation, and reduced opportunities to forage. Integration with an extant population may provide opportunities for social information transfer. However, in the absence of interactions with residents, it is unclear how individual and social learning may affect an animal’s ability to track resources in an unfamiliar landscape. We use integrated step selection functions (iSSFs) to address these knowledge gaps, by evaluating the extent to which environmental factors, individual experience (time since release), and social information-sharing (group size) influence movement decisions by scimitar-horned oryx (*Oryx dammah*) reintroduced into their native range for the first time in ca. 30 years. We found that both experience and social factors influenced the habitat selection and movement behavior of reintroduced oryx. Of four candidate iSSFs, the model that included environmental, experience, and group size variables performed best in both dry and wet periods. Statistically significant interaction terms between environmental variables and experience were generally larger than similar terms for group size, indicating that experience may affect habitat selection by reintroduced oryx more strongly than social factors. These findings may inform the management of recovering wildlife populations, update widely-held expectations about how released ungulates acclimate to novel landscapes, and demonstrate the utility of long-term monitoring of reintroduced populations.

**Supplementary Information:**

The online version contains supplementary material available at 10.1186/s40462-022-00348-z.

## Background

Wildlife reintroductions aim to restore ecosystems by establishing self-sustaining populations of animals within their native range [1, 2]. However, reintroduced animals—especially those raised in captivity—are in the unique and challenging position of navigating a completely unfamiliar environment. They lack important knowledge about the distribution of resources, microclimates, and potential refuges from predators [3, 4]. As a result, reintroduced animals often make erratic or extensive movements upon release [5–17]. While these exploratory movements may increase familiarity with the novel environment [18–20], they can also be energetically costly [21, 22], increase exposure to predation risk [23–25], and reduce opportunities to forage. These combined effects may lower the survival and reproductive success of a reintroduced population, ultimately leading to reduced probability of establishment and long-term viability [26, 27].

Given the potentially high costs of naivety, it is essential for reintroduced individuals to acquire information about their new environment as efficiently as possible. In social species, this process may be facilitated by information transfer among conspecifics. Released bighorn sheep (*Ovis canadensis*), Persian fallow deer and Iberian ibex (*Capra payrenaica*) were strongly attracted to extant groups near their reintroduction sites [28–30]. Such attraction may accelerate reintroduced animals’ acclimation to the novel environment, and decrease the time dedicated to vigilance against predators and conspecific aggression [31, 32]. Conversely, in more solitary species, released animals may be forced to occupy less suitable habitat due to direct [33] or interference [34] competition with established populations or individuals.

There is also mounting evidence that individual experience (defined as time spent in the landscape since release) is a key determinant of the establishment and survival of reintroduced individuals. Berger-Tal and Saltz [18] found that Persian fallow deer (*Dama mesopotamica*) transitioned from slow, short-distance movements near the release site to a bi-modal pattern of movement, indicating exploitation of known resource patches, within a year of release. However, the general expectation that increased experience leads to modified movement strategies is largely based on studies that primarily evaluated either space use (e.g., [17, 28, 35–39]) or assimilation with resident populations (e.g., [29, 30, 32, 40–42]). Few studies have investigated the dynamics of post-release movement behaviors in the absence of interactions with an extant population. As a result, how individual and social learning may contribute to an animal’s ability to track resources in a unfamiliar landscape is not well understood. We use integrated step selection functions (iSSF) to address these knowledge gaps, by evaluating the extent to which environmental factors, individual experience (time since release), and social information-sharing (group size) affect movement decisions by naïve animals reintroduced into their native range for the first time in approximately 30 years.

The scimitar-horned oryx (*Oryx dammah*; hereafter “oryx”) is a large antelope that formerly occupied seasonal grasslands surrounding the Sahara Desert. The species has been classified as Extinct in the Wild since 2000 [43]. A reintroduction project led by the Environment Agency – Abu Dhabi (EAD), the Chadian Ministère de l'Environnement, de la Pêche et du Développement Durable (MEPDD), and implemented by Sahara Conservation (SC), is working to establish a self-sustaining population of oryx in Chad [44]. The first 21 oryx were released into a large, unfenced protected area in 2016. The reintroduced population now exceeds 400 free-roaming animals that are routinely monitored in the field, more than half of which have been tracked using GPS collars.

Because all reintroduced oryx were born and raised in captivity, long-term memory is unlikely to influence their movement behavior or habitat preferences. These conditions offer a unique opportunity to evaluate the influence of social information-sharing among recently reintroduced ungulates on their movement behavior and resource selection. We present an empirical assessment of post-release movements to identify the mechanisms that underly the acclimation of naïve animals to novel environments. These findings may inform the management of recovering wildlife populations, update widely-held expectations about how released ungulates acclimate to novel landscapes, and demonstrate the utility of long-term monitoring of reintroduced populations.

## Methods

### Study species and movement data

Oryx are a large African antelope adapted to the arid, seasonally dynamic steppes characteristic of Sahelian ecosystems. Adults are predominantly white, with rufous coloration on the forehead, neck, and shoulders, and long, curved horns arching over their back. Wild oryx once numbered in the hundreds of thousands, performed seasonal migrations, and ranged across the Sahel from the Atlantic coast to the Red Sea [45–47]. Overhunting was the primary factor in the species’ decline, augmented by regional armed conflicts, habitat degradation and fragmentation, and competition with livestock [48]. The last confirmed sightings of wild oryx occurred in the mid-1980s [47, 49].

During the rainy (ca. July–September) and cool (ca. October–February) periods, oryx primarily foraged on perennial grasses (e.g., *Panicum turgidum*, *Aristida mutabilis*), seed pods (e.g., *Acacia tortilis*), shrubs (e.g., *Cornulaca monacantha*, *Chrozophora senegalensis*, *Cassia italica*), and herbs (e.g., *Heliotropium trigosums*) [47, 50–53]. In wet and cool conditions, oryx are thought to have traveled northwards to seasonally productive grasslands at the edge of the Sahara Desert, largely comprised of annual grasses such as *Cenchrus biflorus*, *Dactyloctenium aegyptiacum*, *Echinochloa colona*, and *Limeum viscosum* [47, 51, 54]. At the peak of the dry season (March–June), oryx are thought to have performed more restricted movements, spending most of their time seeking shade in wooded wadis (areas that temporarily collect water), interdunal depressions, and under isolated trees [47, 50, 51, 53, 55]. The wild melon *Citrullus colocynthis*, succulents, and interdunal depressions that retain green vegetation, like shrubs and young annual plants, may also have been important resources for the species during these times [47, 50–53, 55].

**Fig. 1:**
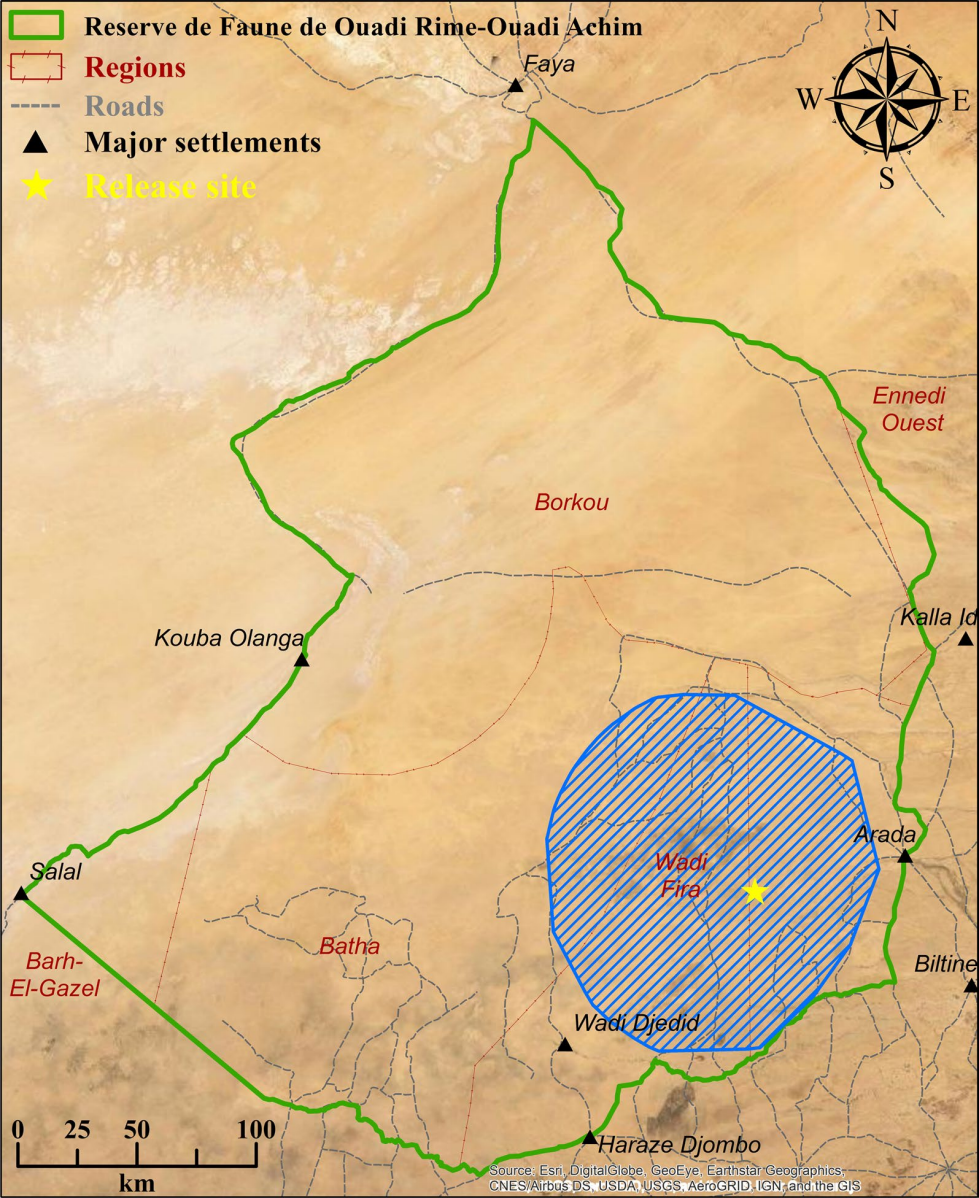
Map of the Réserve de Faune du Ouadi Rimé-Ouadi Achim (RFOROA), including the site where oryx were released (yellow star) and the area where oryx occurred during the study period (blue hatched polygon)

In part due to the enduring importance of oryx in North African and Middle Eastern cultures, large, genetically diverse populations remained in private collections and zoological institutions. The “World Herd” managed by EAD now functions as a source population for restoring the species to the wild. Following a series of stakeholder workshops from 2009 to 2012, and a habitat suitability analysis [56], the ca. 75,000 km^2^ Réserve de Faune du Ouadi Rimé-Ouadi Achim (RFOROA) in central Chad was selected as the target site for oryx restoration (Fig. 1).

In August 2016, after 6 months of acclimating to local conditions in a large (100 ha) enclosures, 21 oryx were released into the RFOROA. Nineteen oryx in the first release group (90%) were fitted with GPS/satellite collars (Vectronic Aerospace GmbH, Berlin, Germany) that collected positions every one (*n* = 17) or four (*n* = 2) hours. In January 2017, 13 additional oryx were released after a shorter, 1-month acclimation period. All oryx in the second release group were fitted with GPS/satellite collars, set to collect positions every one (*n* = 9), two (*n* = 2), or four (*n* = 2) hours. Oryx were collared during brief periods of restraint (< 10 min) in a drop-chute device (Fauna TAMER Jr, Fauna Research Inc., Red Hook, NY, USA). Animal handling methods were approved by the International Animal Care and Use Committee (IACUC) at the Smithsonian Institution and authorized under a cooperative agreement between SCF and MEPDD. Because fix acquisition schedules varied among collared oryx, we resampled all data to a four (4) hour interval. The resulting movement data set consisted of 112,676 locations collected by *n* = 32 collared oryx.

### Study area

The RFOROA was gazetted in 1969 to protect local wildlife, including the currently Critically Endangered dama gazelle (*Nanger dama*) and addax (*Addax nasomaculatus*), the Vulnerable Dorcas gazelle (*Gazella dorcas*), and the locally extinct cheetah (*Acinonyx jubatus*) and African wild dog (*Lycaon pictus*). The reserve contains wooded grasslands, sub-desert grasslands, and desert habitats ranging from 190 to 461 m in elevation, occasionally crossed by wadis and rock outcroppings (Fig. 1). Ephemeral wetlands, primarily the seasonally flooded *Ouadi Kharma* and *Ouadi Achim*, also support migrating and overwintering white storks, ducks, waders, and passerines [57]. While hunting is prohibited within the RFOROA, nomadic pastoralists make extensive use of native vegetation for livestock grazing.

### Environmental and social covariates

We compiled environmental variables considered *a priori* to be important for oryx resource selection and movement (see Additional file 1: Table S1), including anomaly in Normalized Difference Vegetation Index (aNDVI; [58]), dynamic change in NDVI (dNDVI), elevation, topographic complexity, and temperature. NDVI is strongly correlated with vegetation productivity or greenness [59, 60], with positive values indicating vegetation greening and negative values indicating vegetation drying or loss. aNDVI is the difference between the current NDVI measurement and the mean NDVI over a 4-year period (2016–2020), and provides a measure of vegetation greenness relative to longer-term trends [61]. In contrast, dNDVI captures short-term change (16 days) in vegetation greenness (dNDVI = NDVI_t_–NDVI_t-16_). Void-filled shuttle radar topography mission (SRTM) data [62] was used to calculate topographic roughness (TRI; [63]), which characterizes topographic complexity.

All remote sensing variables were resampled via bilinear interpolation from their native resolution to a common resolution of 500 m. Reintroduced oryx routinely moved 100–500 m between hourly GPS fixes, and thus likely encountered multiple 500 m pixels during a 4-h step. An analysis resolution of 500 m was selected as an appropriate balance between the spatial heterogeneity of the study system, the native resolution of environmental covariates, and the movement capacity of the study species at 4-h intervals. All environmental data were extracted and processed in Google Earth Engine [64] and R (version 4.0.3 [65]) using the *raster* package [66].

We also included variables that captured post-release experience and information-sharing among reintroduced oryx. Post-release experience was quantified as the number of days since an individual was released (hereafter “experience”), and was included to assess whether oryx modify movement characteristics or selection for environmental conditions over time. After release, individual animals in a reintroduction cohort may accrue different experiences, such as encountering different resource patches, thermal refugia, or high-risk areas. Individuals may then share this information by attracting (or repelling) social partners or animals in the same social group toward (or away from) these locations of interest. Larger social groups may have access to larger pools of information about locations of interest, and thus may benefit more from the social transfer of information. We quantified the potential for social information-sharing as the total number of oryx in a social group (hereafter “group size”), defined as the set of oryx that were within 200 m of at least one other oryx (i.e., the “chain rule” sensu [67]) for a four (4) hour time-period.

### Integrated step selection analysis

We evaluated oryx resource selection at relatively fine spatio-temporal scales (fourth order selection; [68] using an integrated step selection function (iSSF). We generated nine (9) available steps for each used location, drawing step lengths from a gamma distribution and turning angles from a von Mises distribution fitted to the empirical movement data. This process generated 972,709 used and available steps for n = 32 oryx. Environmental and social covariates extracted from used and available points [69] were centered and standardized to facilitate model convergence and coefficient comparison [70].

We constructed candidate iSSF models for two periods: “dry” (January–June, when virtually no precipitation falls in the study area, and vegetation is largely senescent) and “wet” (July–December, when the vast majority of precipitation falls, and peak vegetation growth occurs), to capture environmental heterogeneity across the study period. Four (4) candidate iSSF models were constructed in each period, containing (1) environmental covariates only, (2) environmental and experience covariates, (3) environmental and group size covariates, and (4) environmental, experience, and group size covariates. We included step as a stratum variable, and step length and log-transformed step length to correct selection estimates [71, 72] and to model their interaction with group size and experience [73]. We also included oryx identity as a cluster variable to account for individual variation in movement patterns, which may otherwise cause bias when making inferences at the population level [74]. Additionally, quadratic terms for short-term vegetation productivity (dNDVI), long-term vegetation productivity (aNDVI) and temperature were included to account for non-linear effects and allow optimum selection. Because group size and experience were constant within strata, we included them as only a one-way interaction term with each covariate of interest for models 2–4 to evaluate how they affected oryx movements and responses to environmental covariates. Formulas for all competing models are included as Additional file 1: Tables S6 and S7. No model covariates exhibited collinearity, as assessed using a Variance Inflation Factor (VIF) analysis (VIF < 4). We used Akaike’s information criterion corrected for small sample sizes (AICc, [75]) to identify the best model for each period. All models were fitted using conditional logistic regression in R using the amt [69] and survival [76] packages.

Finally, we generated model predictions based on the observed range of environmental covariates. Because group size and experience were centered and standardized, and were only included as an interaction term, their predicted values were assigned to three categories: predicted values at least one standard deviation below the mean experience or group size (− 1), the mean experience or group size (0), and predicted values at least one standard deviation above the mean experience or group size (+ 1). Thus, all predicted values at least one standard deviation below the mean category were considered to be a “small” group or “low” experience, predicted values within one standard deviation of the mean were considered to represent “median” group size or experience, and predicted values at least one standard deviation above the mean category were considered “large” groups or “high” experience.

## Results

Both experience and group size influenced habitat selection and movement behavior of reintroduced oryx. Of four candidate iSSFs, the model including environmental, experience, and group size variables (M4) performed best in both dry and wet periods (see Additional file 1: Tables S2 and S3, and Figures S1 and S2). Model 4 also included interaction terms to evaluate how experience and group size influenced oryx responses to environmental factors (Table 1). Statistically significant interaction terms between environmental variables and experience were generally larger than statistically significant interaction terms between environmental variables and group size, indicating that post-release experience may affect habitat selection by reintroduced oryx more strongly than social information.

**Table 1:** Coefficient estimates, standard errors (SE), and relative selection strengths (RSS) for the final integrated step selection functions (iSSFs) for reintroduced oryx during dry and wet periods

Variable	Dry period			Wet period		
	Coefficient	RSS	SE	Coefficient	RSS	SE
Step length	0.031216	1.031709	0.006206	− **0.085392**	**0.918152**	**0.007023**
Log step length	− **0.066014**	**0.936118**	**0.006020**	**0.145373**	**1.156471**	**0.007344**
aNDVI	**0.220561**	**1.246776**	**0.025554**	**0.040974**	**1.041825**	**0.012642**
dNDVI	− **0.118859**	**0.887933**	**0.040020**	**0.034369**	**1.034966**	**0.015879**
Elevation	**0.033046**	**1.033598**	**0.007553**	− **0.028966**	**0.971450**	**0.009258**
TRI	− **0.069324**	**0.933025**	**0.005778**	**0.027761**	**1.028150**	**0.005825**
Temperature	− **0.018336**	**0.981831**	**0.008080**	**0.008782**	**1.008821**	**0.007739**
Experience	0.013913	1.014010	0.032343	− 0.012551	0.987527	0.029001
Group size	− 0.002478	0.997526	0.005738	− **0.007034**	**0.992991**	**0.005433**
I(aNDVI^2^)	− **0.018772**	**0.981403**	**0.003777**	− **0.010235**	**0.989817**	**0.003493**
I(dNDVI^2^)	− **0.009814**	**0.990234**	**0.003132**	− **0.009695**	**0.990352**	**0.004679**
I(Temperature^2^)	− **0.012278**	**0.987797**	**0.005011**			
Step length: experience	0.002168	1.002170	0.005965	**0.031899**	**1.032414**	**0.006149**
Log step length: experience	− **0.035228**	**0.965386**	**0.005695**	− 0.032891	0.967644	0.006980
aNDVI: experience	0.033742	1.034317	0.031870	0.013910	1.014007	0.008011
dNDVI: experience	− **0.160548**	**0.851677**	**0.051272**	− 0.009679	0.990368	0.013125
Elevation: experience	− **0.033265**	**0.967283**	**0.009731**	**0.020697**	**1.020912**	**0.010874**
TRI: experience	**0.052383**	**1.053779**	**0.006479**	− **0.015166**	**0.984949**	**0.007235**
Temperature: experience	**0.031204**	**1.031696**	**0.010877**			
Step length: group size	− **0.121270**	**0.885795**	**0.007104**	− **0.057897**	**0.943747**	**0.007664**
Log step length: group size	**0.102707**	**1.108167**	**0.007067**	**0.072373**	**1.075057**	**0.008066**
aNDVI: group size	0.003632	1.003638	0.004126	**0.007308**	**1.007335**	**0.006514**
dNDVI: group size	**0.004128**	**1.004136**	**0.004067**	− **0.003953**	**0.996055**	**0.005836**
Elevation: group size	0.007185	0.992841	0.006904	− **0.014649**	**0.985458**	**0.006387**
TRI: group size	**0.026482**	**1.026836**	**0.005851**	**0.016766**	**1.016908**	**0.005480**
Temperature: group size				**0.000303**	**1.000303**	**0.005580**

Bold text indicates significance at the *p* < 0.05 threshold

### Dry period

**Fig. 2:**
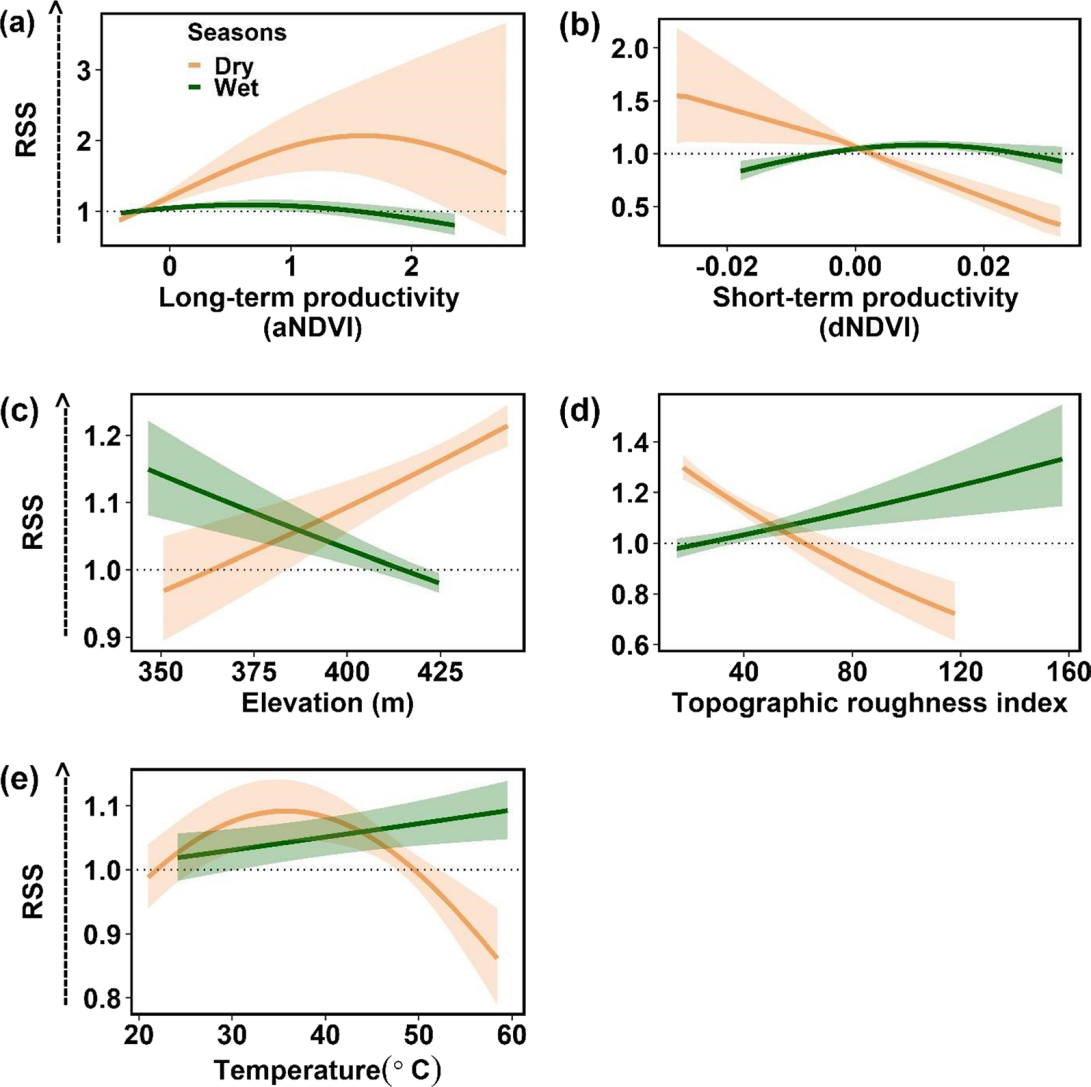
Oryx relative selection strength (RSS) for environmental conditions during the dry (brown) and wet (dark green) periods. Environmental covariates include: **a** long-term vegetation productivity (aNDVI), **b** short-term vegetation productivity (dNDVI), **c** elevation, **d** topographic roughness index, and **e** temperature. Horizontal dotted line indicates neither selection nor avoidance, values > 1 indicate selection for (preference) and values < 1 indicate selection against (avoidance). Shading indicates 95% confidence intervals. Detailed descriptions of environmental covariates may be found in Additional file 1: Table S1

At the population level, and across all experience and group size values, reintroduced oryx strongly selected sites with elevated long-term vegetation productivity (aNDVI; Fig. 2a) during the dry period, yielding a positive, hump-shaped response to aNDVI (Table 1). In contrast, reintroduced oryx exhibited a negative, relatively linear response to short-term vegetation productivity (dNDVI; Fig. 2b). Reintroduced oryx also generally selected sites with locally high elevation and low topographic complexity (TRI; Fig. 2c, d).

**Fig. 3:**
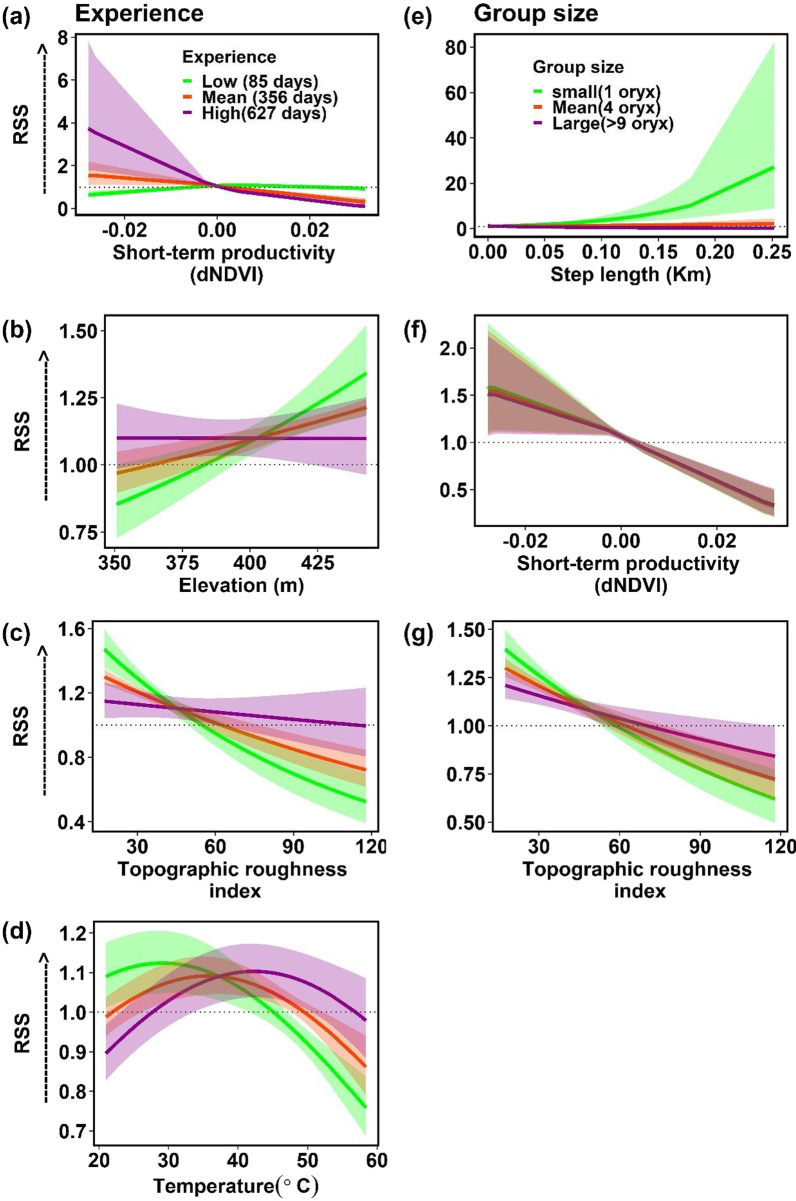
Interactions between experience, group size, and environmental conditions in the final dry period model. Significant interactions between experience and environmental covariates in the final dry period model included: **a** Short-term vegetation productivity (dNDVI), **b** elevation, **c** topographic roughness index, and **d** temperature. Significant interactions between group size and other covariates included: **e** step length, **f** short-term vegetation productivity (dNDVI), **g** Topographic roughness index. Horizontal dotted line indicates neither selection nor avoidance, values > 1 indicate selection for (preference) and values < 1 indicate selection against (avoidance). Shading indicates 95% confidence intervals. Detailed descriptions of environmental covariates may be found in Additional file 1: Table S1

As reintroduced oryx gained experience, their tolerance for short-term vegetation drying and loss during the dry period substantially increased. While oryx with low and median levels of experience (ca. 3 months and ca. 1 year roaming the RFOROA, respectively) showed a relatively flat response to dNDVI, the most experienced oryx (at least 18 months in the RFOROA) exhibited a much steeper negative response to dNDVI (Fig. 3a), particularly at negative dNDVI values. At the same time, more experienced oryx selected sites relatively evenly across available topographic conditions (elevation and TRI), indicating that reintroduced oryx become increasingly indifferent to topographical conditions during the dry period over time (Fig. 3b, c).

Unexpectedly, we found that experienced oryx are also increasingly tolerant of higher temperatures (Fig. 3d). During the dry period, oryx within 3 months of release exhibit steep negative selection for temperatures above the period-level mean. However, oryx with greater experience exhibit wider, flatter response curves to temperature, indicating a gradual increase in thermal tolerance over the time since release.

Most interactions between environmental variables and group size were relatively small (Table 1), with high overlap across group sizes (Fig. 3g, h). However, oryx moving alone during the dry period were more likely to avoid areas with high topographic complexity (Fig. 3f) compared to larger groups (more than 9 animals). Oryx traveling alone were also more likely to take longer steps, and exhibited much more variation in step lengths, than oryx traveling in groups (Fig. 3e).

### Wet period

During the wet period, oryx selected sites with intermediate long-term vegetation productivity and elevated short-term vegetation productivity, indicated by hump-shaped relationships with aNDVI and dNDVI (Fig. 2). While these effects were statistically significant, relative selection strength for aNDVI was weaker, and the estimated coefficients for both vegetation productivity variables smaller, than in the dry period (Table 1). Larger groups of oryx (n ≥ 7) exhibited stronger selection for sites with higher aNDVI and dNDVI than smaller groups, while interactions between aNDVI or dNDVI and experience were not significant.

**Fig. 4:**
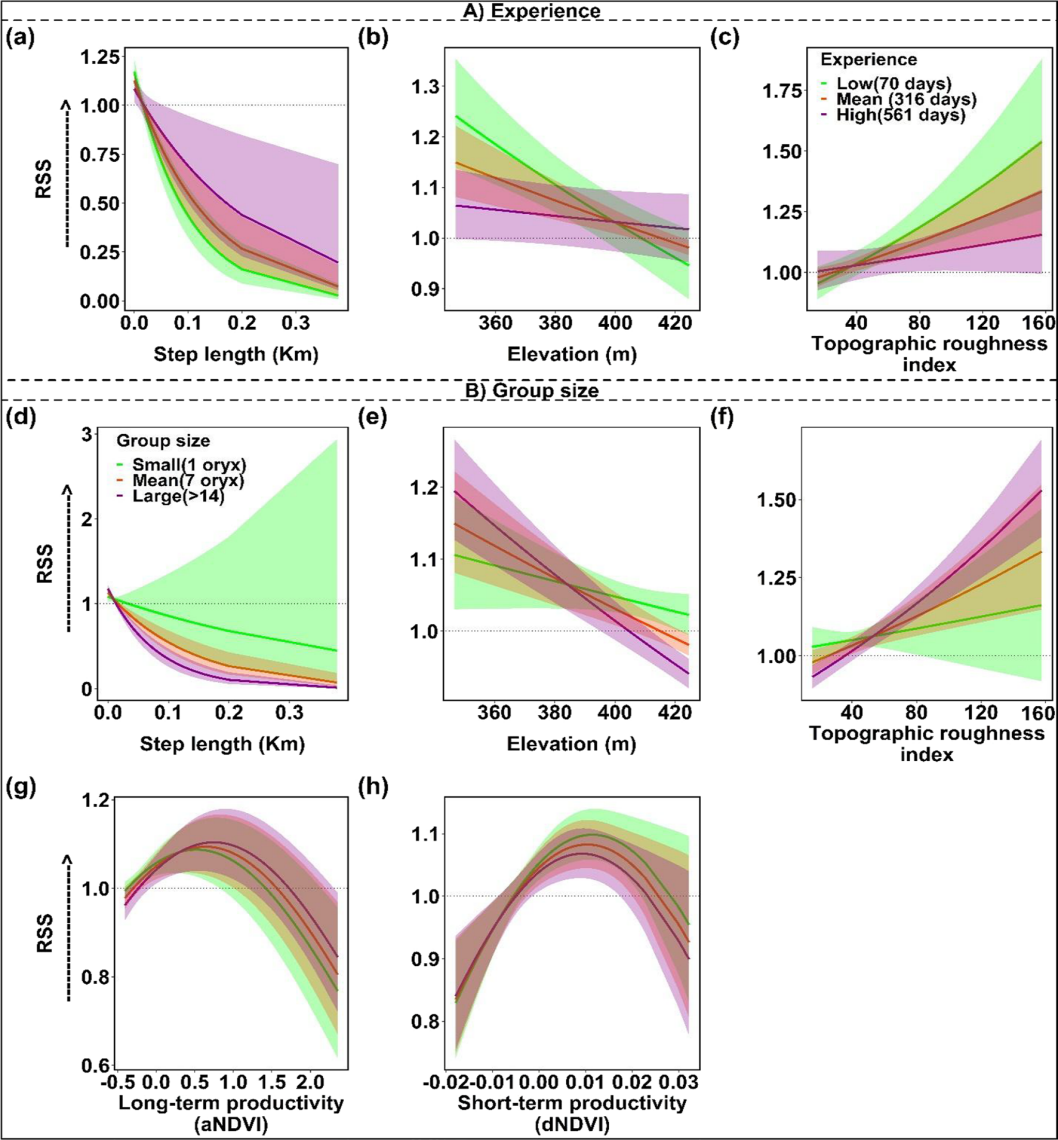
Significant interactions between covariates in the final wet period model. Significant interactions between experience and environmental covariates in the final wet period model included: **a** step length, **b** elevation, and **c** Topographic roughness index. Significant interactions between group size and other covariates included: **d** step length, **e** elevation, **f** topographic roughness index, g) Long-term vegetation productivity (aNDVI), and h) Short-term vegetation productivity (dNDVI). Horizontal dotted line indicates neither selection nor avoidance, value > 1 indicates selection for (preferences) and value < 1 indicates selection against (avoidance). Shading indicates 95% confidence intervals. Detailed descriptions of environmental covariates may be found in Additional file 1: Table S1

Interestingly, experience and social context had disparate effects on oryx responses to topographic factors during the wet period. Overall, oryx exhibited statistically significant preferences for sites with low elevation and high TRI during the wet period (Table 1; Fig. 2c, d). These preferences are consistent with the exploitation of inter-dunal depressions, which collect moisture and thus often support trees and patches of productive grasses. Preferences for these topographic conditions faded as animals gained experience (Fig. 4b, c). However, larger group sizes exhibited a steeper negative response to elevation and a steeper positive response to TRI (Fig. 4e, f), revealing stronger preferences for inter-dunal resource patches by larger groups of oryx, compared to smaller groups.

Similar to the dry period model, oryx traveling alone generally took longer steps—and steps of more variable length—than oryx traveling in groups (n ≥ 7; Figs. 3e, 4d). However, oryx with more experience significantly preferred longer steps in the wet period (Fig. 4a)—indicating that experienced oryx are more likely to take longer steps when resources are comparatively abundant. Lastly, oryx exhibited a response to temperature that was both positive and nearly linear, presenting both a weaker response than that observed in the dry period model, and strongly indicating reduced heat stress during this period.

## Conclusions

This is the first study to evaluate the potentially interacting influences of environmental factors, individual experience (time since release), and social information transfer (group size) on the movement behavior and resource selection of reintroduced ungulates. While experience and social factors are recognized as important for the success of reintroduced populations, they are rarely explicitly included in analyses of movement behavior or resource selection. Translating these ecological concepts into discrete metrics, and explicitly including them in analyses of post-release behavior, offers useful information for interpreting the movements of reintroduced animals in a novel environment. Our primary interest was population-level insights for scimitar-horned oryx reintroduced into Chad; thus, we included data from multiple groups of oryx released within months of each other, and interpreted model results across all individuals.

Historical observations of wild oryx suggested that reintroduced oryx would exploit forage that may persist in inter-dunal depressions during dry periods [47, 50–53, 55]. Contrary to these expectations, our analysis showed that reintroduced oryx preferred inter-dunal depressions only during the wet period. This unexpected result may be due to the extensive changes in both suitable habitat—i.e., loss or degradation of inter-dunal depressions—and human activity—i.e., increased competition with humans and livestock for access to depressions—since the species’ extinction in the wild during the 1980s. These relationships also suggest that reintroduced oryx conserve energy during the dry period, by avoiding rough terrain and limiting effort spent searching for rare, isolated resource patches of uncertain quality. An energy conservation strategy is an intuitively advantageous tactic during dry periods in the Sahelian grasslands, when resource availability is highly limited. We also found that preferential selection for inter-dunal depressions weakened with greater experience, suggesting that, once oryx have gained familiarity with the study landscape, intensive exploitation of inter-dunal depressions is not necessary for survival.

One exception to this pattern may be large groups of oryx (n > 14), which exhibited a steeper negative response to elevation, a steeper positive response to topographic complexity, and selected higher values of aNDVI during the wet period, compared to smaller groups. Several mechanisms could explain these contrasting responses. First, larger groups have greater overall caloric requirements, and potentially more intense competition for food among group members. Thus, large groups may be more dependent on exploiting resource patches to maintain group cohesion. Second, larger groups may have higher search efficiency. More individuals, searching across a larger area—and moving relatively slowly, as indicated by significant, negative interactions between group size and step length in both wet and dry periods (see Table 1)—may better detect resource patches than smaller groups. Finally, larger groups may have access to a larger pool of information about resource patches—i.e., the accumulated memories of resource encounters across all group members. The social transfer of information about patch location and quality among oryx in large groups may offset otherwise untenable energy costs required to travel between rare and isolated patches.

Small groups of oryx tended to take longer steps than large groups, in both dry and wet periods. This finding is likely due to the different, and potentially diverse, motivations of oryx moving alone, compared with oryx moving as a group. For example, three oryx repeatedly engaged in solo long-distance movements during the study period. These movements were distinct from the movements of large groups across multiple movement metrics (e.g., daily and total distance traveled, net displacement from the release site, and mean step length), and likely represent exploration, prospecting, or mate-searching behaviors, which tend to be exhibited by single individuals or small groups. Moreover, excluding these three individuals from analysis did not substantially alter our iSSF results.

More experienced reintroduced oryx appeared to tolerate higher temperatures over time. Similar to other desert-adapted species [77–79], this apparent gain in thermal tolerance most likely arose because more experienced oryx exploited cooler habitats than inexperienced oryx. During the hottest months of the year (approximately April–June), many oryx rest during daytime hours, and move at night, when temperatures are comparatively cool (T. Wacher, *unpublished data*). These model outcomes and in situ observations indicate that reintroduced oryx adopted one movement strategy upon release, then performed a behavioral modification: a promising development in a founder population that has spent many generations in captivity.

Because we were primarily interested in population-level inference, we did not assess inter-individual variation. However, this approach may be useful in future studies, to assess intraspecific differences in oryx post-release movement strategies and resource selection. All models were constructed at 500 m as a balance between regional environmental structure, the native resolution of remote sensing data, and species-specific expectations for perceptual range and movement capacity. Future analyses could test different analysis grains to explicitly estimate and construct models at the study species’ response grain. In addition, the stronger preference for inter-dunal resource patches by large groups was somewhat weak; a longer post-release tracking period, or data from additional releases, are necessary to further investigate potential relationships between group size and patch exploitation.

This study found that scimitar-horned oryx reintroduced into their native range after many generations in captivity adopted movement strategies suitable for a completely novel and seasonally extreme environment. Integrated step selection functions (iSSFs) indicated that reintroduced oryx exploited inter-dunal resource patches during wet periods, and avoided rough terrain and inefficient search behaviors during dry periods, likely conserving energy when forage is seasonally limited. Reintroduced oryx also showed decreasing preferences for inter-dunal depressions, and increasing tolerance for both higher temperatures and topographical conditions, with experience, indicating capability for acclimatation to Sahelian grasslands over time. Promisingly, these outcomes occurred at the population level, across both sexes and multiple release groups. In addition, the signal that large groups may more effectively detect and exploit resource patches suggests that integrating recently and previously released oryx may accelerate the processes of acclimation and gaining knowledge about a novel environment. Intensive monitoring of reintroduced animals was critical to assess these post-release dynamics, and remains essential for further evaluating movement behavior and resource selection strategies by reintroduced animals.

## Supplementary Information

**Additional file 1:** Supplementary Materials.

## Data Availability

The movement data analyzed in this study are not publicly available due to the extreme conservation status and ongoing threats to the study species, but may be available from the corresponding author upon reasonable request.
