# Supplementary material for: Experience and social factors influence movement and habitat selection in scimitar-horned oryx (Oryx dammah) reintroduced into Chad
Source: Mov Ecol. 2022 Nov 10;10:47. doi: 10.1186/s40462-022-00348-z (PMC9650856; doi:10.1186/s40462-022-00348-z)
Supplement: Supplementary file 1 — Additional file 1: Supplementary Materials. [file 40462_2022_348_MOESM1_ESM.docx]

**Supplementary Materials**

**Table S1.** Description of environmental variables that were considered for inclusion in integrated step selection function models.

| **Variable** | **Description** | **Source** | **Included / Not included** |
| --- | --- | --- | --- |
| **Anomaly NDVI (aNDVI)** | The difference between the current NDVI and the long-term mean (4 years period (2016-2020), providing a measure of relative greenness in relation to relatively long-term temporal trends. And it is less likely to be influenced by vegetation structure. | - NASA MOD13Q1 - resampled from 250m to 500m - [https://search.earthdata.nasa.gov/](https://www.researchgate.net/deref/https%3A%2F%2Fsearch.earthdata.nasa.gov%2F) | Included |
| **dNDVI** | dynamic change in vegetation greenness between the current NDVI value and the previous NDVI value (dNDVI = NDVI_t_ – NDVI_t-1_), NDVI is known to be strongly correlated with a location’s vegetation productivity/greenness. | - NASA MOD13Q1 - resampled from 250m to 500m - [https://search.earthdata.nasa.gov/](https://www.researchgate.net/deref/https%3A%2F%2Fsearch.earthdata.nasa.gov%2F) | Included |
| **Elevation** | Mean altitude above the sea levels in Meters. | - Elevation data acquired by the Shuttle Radar Topography Mission (SRTM) and void-filled by the US National Geospatial Intelligence Agency via interpolation - resampled from 30m to 500m - <https://doi.org/10.5066/F7PR7TFT> | Included |
| **Topographic roughness index** | Topographic complexity of the landscape. Small values indicate low variability, and large values indicate high complexity. Calculated as the square root of the squared sums of the differences in elevation from a center cell and its eight surrounding cells. | - Calculated from SRTM data using the method proposed by Riley et al. (1999) - resampled after calculation from 30m to 500m | Included |
| **Temperature** | Measure of the degree of hotness or coldness of the land surface. This may affect resources availability directly and indirectly, and at the same time likely to affects the oryx movement. | - NASA MOD11A2 - resampled from 1km to 500m - [https://search.earthdata.nasa.gov](https://www.researchgate.net/deref/https%3A%2F%2Fsearch.earthdata.nasa.gov%2F) | Included |
| **Experience** | Maximum number of days oryx have been in the landscape since being released into the wild. | Calculated from the date each group of oryx was released into the Reserve de Ouadi Rime-Ouadi Achim | Included |
| **Group size** | Total number of oryx within at least 200 meters of each other during a four-hour period. | Calculated from GPS collar data | Included |
| **Precipitation** | Daily amount of precipitation in mm | - Gridded cumulative rainfall data produced by the Climate Hazards Group InfraRed Precipitation with Station data (CHIRPS) - 0.05° resampled to 500m - <https://www.chc.ucsb.edu/data/chirps> - see Funk et al. (2015) | Not included, because the amount of precipitation received in this region is relatively low with little spatial variation and lots of zeros (0) |
| **Topographic wetness index** | Metric used for determining the capacity of the landscape to support vegetation growth. | - Calculated from SRTM elevation data - resampled from 30m to 500m | Not included |
| **Landscape curvature** | Topographic ups and downs of the landscape. The value ranges from 1 indicating hilltop to -1 indicating valley and 0 flat areas of the landscape. | - Calculated from SRTM elevation data using the *curvature* function from the *spatialEco* package in R - resampled from 30m to 500m | Not included (topographic structure is relatively uniform, with some high dunes and inter-dunal depressions. |

  
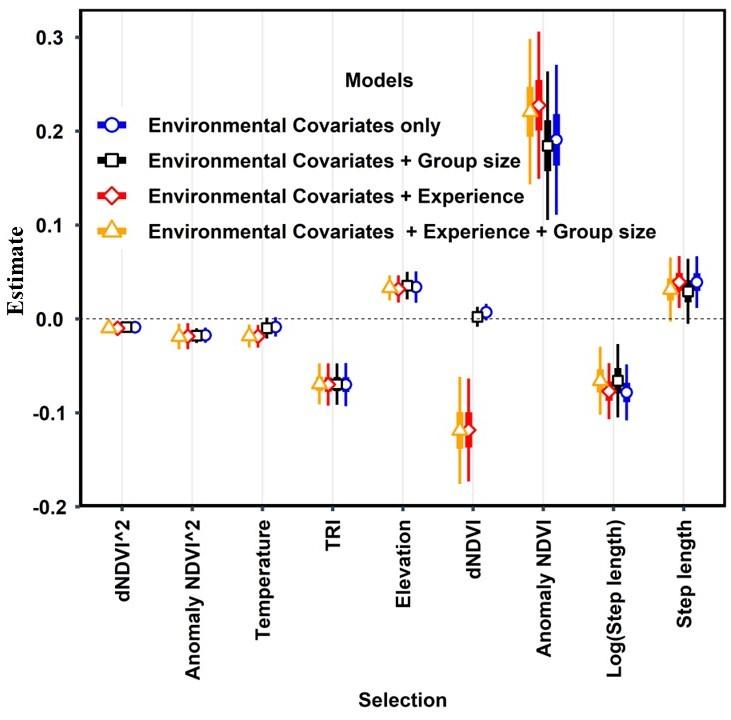


**Figure S1.** Competition and evaluation of four potential models of oryx movement and resource preferences during the dry period (*models described in figure legend*). Error bars indicate 95% confidence intervals.


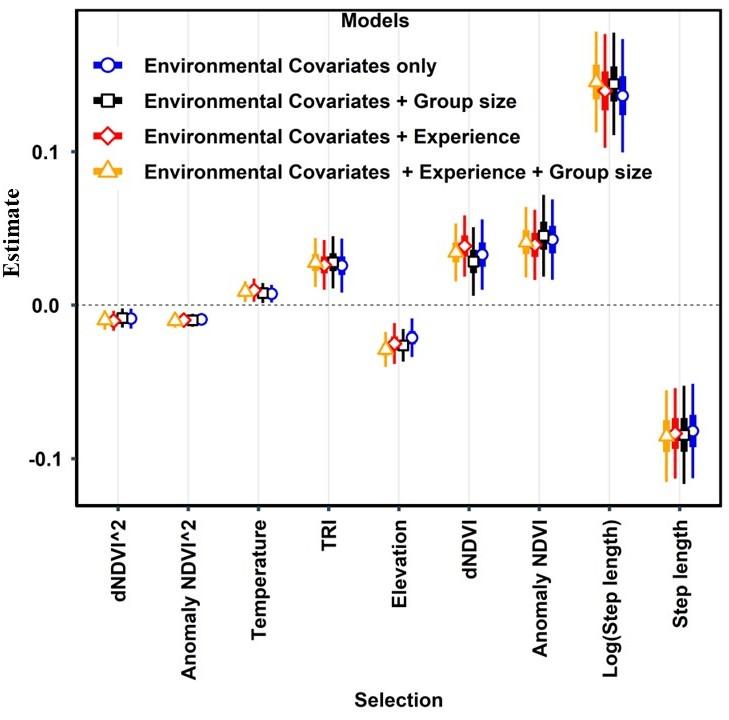


**Figure S2.** Competition and evaluation of four potential models of oryx movement and resource preferences during the wet period (*models described in figure legend*). Error bars indicate 95% confidence intervals.

**Table S2:** Structures of the four candidate iSSFs models during the dry period ranked by their AIC(AICc) and Akaike weight (AICcWt).

| **Model structure** | **K** | **AICc** | **Delta AICc** | **AICcWt** | **LL** |
| --- | --- | --- | --- | --- | --- |
| Environmental covariates + group size + experience (M4) | 25 | 455901.9 | 0 | 1 | -227925.9 |
| Environmental covariates + group size (M2) | 18 | 456025.1 | 123.2406 | 0 | -227994.6 |
| Environmental covariates + experience (M3) | 18 | 456247.2 | 345.2970 | 0 | -228105.6 |
| Environmental covariates only (M1) | 10 | 456397.2 | 495.2660 | 0 | -228188.6 |

**Table S3: S**tructures of the four candidate iSSFs models ranked by their AIC(AICc) and Akaike weight (AICcWt) during the wet period.

| **Model structure** | **K** | **AICc** | **Delta AICc** | **AICcWt** | **LL** |
| --- | --- | --- | --- | --- | --- |
| Environmental covariates + group size + experience (M4) | 24 | 451117.6 | 0 | 0.9999943 | -225534.8 |
| Environmental covariates + group size (M2) | 17 | 451141.7 | 24.15608 | 5.682927e-06 | -225553.9 |
| Environmental covariates + experience (M3) | 17 | 451201.0 | 83.45917 | 0 | -225583.5 |
| Environmental covariates only (M1) | 9 | 451260.5 | 142.96256 | 0 | -225621.3 |

**Table S4**. Summary of the covariates used for integrated step selection models during dry season. The used and available step values in the table below are prior to standardization.

| Covariate | Used step (Mean and range) | Available step (Mean and range) |
| --- | --- | --- |
| dNDVI | -0.0007447 (-0.02773– 0.03075) | -0.0007427(-0.02775 –0.03192) |
| aNDVI | -0.1500 (-0.4168 – 2.6095) | -0.1530 (-0.4184 – 2.7818) |
| Elevation | 396.8 (352.5 – 437.1) | 396.7 (350.8 – 443) |
| Topographic roughness index | 47.88 (20.02 – 117.16) | 48.46 (17.41 – 117.85) |
| Temperature | 39.58 (22.51 – 57.83) | 39.57 (20.99 – 58.41) |
| Experience | 355.7 (1 – 1418) days | 355.6 (1 – 1418) days |
| Group size | 4 (0 – 25) | 4 (0 – 25) |

**Table S5**. Summary of the covariates used for integrated step selection models during wet season. The used and available step values in the table below are prior to standardization.

| Covariate | Used step (Mean and range) | Available step (Mean and range) |
| --- | --- | --- |
| dNDVI | 0.0006928 (-0.0156348– 0.0315432) | 0.0006837 (-0.0178930 – 0.0322168) |
| aNDVI | 0.005897 (-0.332631 – 2.328095) | 0.004706 (-0.408957 – 2.355096) |
| Elevation | 390.9 (351.3– 421.6) | 391 (346.6 – 424.6) |
| Topographic roughness index | 47.64 (16.88– 157.52) | 47.44 (15.26 – 157.52) |
| Temperature | 39.49 (24.81– 57.83) | 39.47 (24.13– 59.53) |
| Experience | 315.3 (1 – 1511) days | 315.3 (1 – 1511) days |
| Group size | 7 (0 – 31) | 7 (0 – 31) |

**Table S6.** **Competitive model structure**. Competitive model structure for each integrated step selection model used to evaluate movement and habitat selection of scimitar horned oryx reintroduced into Chad during dry season.

| Model Name | Model structures |
| --- | --- |
| Environmental covariates only | M1<-clogit (case_~ Step length + log (step length) + aNDVI + dNDVI+ Elevation + TRI + Temperature + I(aNDVI ^2) + I(dNDVI ^2)+ I(Temperature^2) + cluster(Animal_id) + strata(step_id_), x=TRUE, y=TRUE, method = "efron", model=TRUE, data=Dry) |
| Environmental covariates + group size | M2<-clogit (case_~ Step length + log (step length) + aNDVI +dNDVI+ Elevation + TRI + Temperature + I(aNDVI ^2) + I(dNDVI ^2) + I(Temperature^2) + Group size*( Step length + log (step length) + aNDVI +dNDVI+ Elevation + TRI + Temperature ) + cluster(Animal_id)+ strata(step_id_), x=TRUE, y=TRUE, method = "efron", model=TRUE, data=Dry) |
| Environmental covariates + experience | M3<- clogit (case_~ Step length + log(step length) + aNDVI + dNDVI+ Elevation + TRI + Temperature + I( aNDVI ^2) + I( dNDVI ^2) + I(Temperature^2) + experience*( Step length + log (step length) + aNDVI + dNDVI + Elevation + TRI + Temperature) + cluster(Animal_id) + strata(step_id_), x=TRUE, y=TRUE, method = "efron", model=TRUE, data=Dry) |
| Environmental covariates + group size + experience | M4<-clogit (case_~ Step length + log (step length) + aNDVI + dNDVI + Elevation + TRI + Temperature + I( aNDVI ^2) + I( dNDVI ^2) + I(Temperature^2) + experience*( Step length + log (step length) + aNDVI + dNDVI + Elevation + TRI + Temperature) + Group size*( Step length + log (step length) + aNDVI + dNDVI + Elevation + TRI + Temperature ) + cluster(Animal_id) + strata(step_id_), x=TRUE, y=TRUE, method = "efron", model=TRUE, data=Dry) |

**Table S7.** **Competitive model structure**. Competitive model structure for each integrated step selection model used to evaluate movement and habitat selection of scimitar horned oryx reintroduced into Chad during wet season.

| Model Name | Model structures |
| --- | --- |
| Environmental covariates only (M1) | M1<-clogit (case_~ Step length + log (step length) + aNDVI + dNDVI+ Elevation + TRI + Temperature + I(aNDVI ^2) + I(dNDVI ^2) + cluster(Animal_id) + strata(step_id_), x=TRUE, y=TRUE, method = "efron", model=TRUE, data=Wet) |
| Environmental covariates + group size (M2) | M2<-clogit (case_~ Step length + log (step length) + aNDVI +dNDVI+ Elevation + TRI + Temperature + I(aNDVI ^2) + I(dNDVI ^2) + Group size*(Step length + log (step length) + aNDVI +dNDVI+ Elevation + TRI + Temperature) + cluster(Animal_id)+ strata(step_id_), x=TRUE, y=TRUE, method = "efron", model=TRUE, data=Wet) |
| Environmental covariates + experience (M3) | M3<-clogit (case_~ Step length + log (step length) + aNDVI + dNDVI+ Elevation + TRI + Temperature + I(aNDVI ^2) + I(dNDVI ^2) + experience*(Step length + log (step length) + aNDVI + dNDVI + Elevation + TRI + Temperature) + cluster(Animal_id) + strata(step_id_), x=TRUE, y=TRUE, method = "efron", model=TRUE, data=Wet) |
| Environmental covariates + group size + experience (M4) | M4<-clogit (case_~ Step length + log(step length) + aNDVI + dNDVI + Elevation + TRI + Temperature + I( aNDVI ^2) + I( dNDVI ^2) + experience*( Step length + log(step length) + aNDVI + dNDVI + Elevation + TRI ) + Group size*( Step length + log(step length) + aNDVI + dNDVI + Elevation + TRI + Temperature ) + cluster(Animal_id) + strata(step_id_), x=TRUE, y=TRUE, method = "efron", model=TRUE, data=Wet) |
